# Biochemical parameters, oxidative stress biomarkers, and anatomopathological changes in Wistar rats treated with 3′-demethoxy-6-O-demethylisoguaiacin and norisoguaiacin

**DOI:** 10.1038/s41598-024-61903-9

**Published:** 2024-05-21

**Authors:** Nancy Guadalupe Flores Jiménez, Martha Manzano Zamorano, Guillermo Reséndiz-González, Crisóforo Mercado-Márquez, José Francisco Morales-Álvarez, Jorge Alfredo Cuéllar-Ordaz, Héctor Alejandro de-la- Cruz Cruz, Olivia Adams-Vázquez, María Eugenia López-Arellano, Roberto Díaz-Torres, Patricia Ramírez Noguera, Rosa Isabel Higuera-Piedrahita

**Affiliations:** 1 Facultad de Estudios Superiores Cuautitlán, Universidad Nacional Autónoma de México, Mexico, Estado de México Mexico; 2 Centro Nacional de Investigación Disciplinaria en Salud Animal e Inocuidad, Instituto Nacional de Investigaciones Forestales, Agrícolas y Pecuarias, Palo Alto, Mexico Mexico; 3 Centro Nacional de Investigación Disciplinaria en Salud Animal e Inocuidad, Instituto Nacional de Investigaciones Forestales, Agrícolas y Pecuarias, Jiutepec, Morelos Mexico

**Keywords:** Lignans, Biochemical parameters, Oxidative stress biomarkers, Anatomopathological changes, *Artemisia cina*, Structural biology, Biochemistry, Biological techniques, Cell biology, Chemical biology, Drug discovery, Biomarkers

## Abstract

*Artemisia cina* (*Ac*) is a plant with anthelmintic compounds such as 3′-demethoxy-6-O-demethylisoguaiacin (D) and norisoguaiacin (N)*.* Three major objectives were proposed: (1) To evaluate biochemical parameters in blood (2) to determine the tissue oxidative stress by biomarkers as TBARS and glutathione peroxidase activity, and (3) to evaluate anatomopathological changes in organs such as the brain, liver, kidney, and lung after oral administration of *n-*hexane extract of *Ac* and D and N. D and N were administrated following the OECD guides for acute oral toxicity evaluation (Guide 420). Fifty Wistar rats were distributed into ten groups as follows: Group 1 (G1): 4 mg/Kg; G2: 40 mg/Kg; G3: 240 mg/Kg; G4: 1600 mg/Kg of *n-*hexane extract of *Ac*. G5: 2 mg/Kg; G6: 20 mg/Kg; G7: 120 mg/Kg; G8: 800 mg/Kg of D and N, G9: water and G10: polyvinylpyrrolidone at 2000 mg/Kg. At 14 days, the rats were euthanized, and the blood, liver, brain, kidney, and lung were taken for biochemical analysis, anatomopathological changes, and TBARS and GSH evaluation. Glucose, cholesterol, and phosphorus were altered. Histopathological analysis showed multifocal neuronal degeneration in the brain (G2). The kidney and lungs had changes in G7. The GSH and TBARS increased in G6 and G7. The TBARS activity was higher in G1 and G2. In conclusion, extract and D and N of* Ac* did not have damage at therapeutic doses. *D*, *N*, and *n*-hexane extract of *A. cina* do not cause histopathological damage at pharmaceutical doses. Still, the brain, kidney, and liver are related to biochemical parameters at higher doses. However, compounds are proposed as antioxidant agents.

## Introduction

Anthelmintic drug searches are the most critical activity after resistance reports. In ruminants, *Haemonchus contortus* is a hematophagous parasite that causes significant losses of around million dollars annually^1^. Clinical signals caused by *Haemonchus* infections cause anemia, worse digestion, or death^2^.

The integrated control of parasites includes copper needles, nematophagous fungi, homeopathy, or herbology^3^. Chemical compounds isolated from plants or organic extracts have potential activity against free health or endogenous phases^4^. The activity can differ according to the synergism or additivity of compounds of extracts^5^. *Artemisia* (Asteraceae) is a plant used as an anti-inflammatory, anticancer, and antimicrobial and reported in pharmacopeia as an adjuvant related to human parasitic diseases^6^. Asteraceae family are 200 to 400 species from Asia, Europe, and North America^7^. *Artemisia cina*, with an aromatic smell, belongs to the Asteraceae family from the Caspian Sea in Afghanistan^8^. *A. cina* grows in the desert with saline floor and is used in traditional medicine as an antiparasitic in humans^9^. From 1950 to 1970, the *A. cina* was used as a child antiparasitic for the presence of santonin (an anthelmintic compound)^10^. 1971, artemisinin was reported on the plant as an antimalarial compound^10^. *A. cina* aqueous extract was reported as an antiparasitic agent over *Moniezia expansa*^11^. Ethanolic extract of *A. cina* was reported as an anthelmintic against eggs and infective larvae of *H. contortus*^12^. At the same, *n-*hexane extract of *A. cina* was reported as anthelmintic over natural infections of nematodes in goats^13^. The *n*-hexane extract shows egg per gram reductions of 20.1% after seven days of oral treatment^12^. *A. cina* ethanolic extract, *n*-hexane extract, and ethyl acetate extract showed anthelmintic activity against eggs, infective and hematophagous larvae and adults of *H. contortus* in infected gerbils, artificially infected sheep, naturally infected goats, and in vitro assays^12^. The bio-guided assays showed that the molecules responsible for the effect are 3′-demethoxy-6-O-demethylisoguaiacin and norisoguaiacin separated and isolated from an *n*-hexane extract of *A. cina*. The compounds showed 100% mortality against infective larvae of *H. contortus*^14^.

Both molecules are lignans with antiviral and antibacterial activity. 3′-demethoxy-6-O demetil isoguaiacin is used as an alternative for *Staphylococcus aureus* multi-resistant^15^. Norisoguaiacin showed anti-VIH activity, a significant disease for humans and public health^16^. Also, the molecule has antiprotozoal effects reported against *Trypanosoma brucei rhodesiense, Trypanosoma cruzi, Leishmania donovani, and Plasmodium falciparum*. The last compound also had an antioxidant effect^17^.

Garza-González et al.^18^ reported the activity of molecules separately, and the biological activity in the antibacterial effect was patented^18^. However, the toxicity of the lignans has not been reported when administered orally. The synergistic effect of 3′-demethoxy-6-O-demethylisoguaiacin and Norisoguaiacin against *H. contortus* was proposed as an anthelmintic drug for use in ruminants^14^. However, it is essential to determine the potential acute toxic effect after administration orally. This study aimed to determine the oral acute toxicity of *n*-hexane extract and the mixture of 3′-demethoxy-6-O-demethylisoguaiacin and norisoguaiacin to propose it as a safe anthelmintic.

In the present study, three significant objectives were proposed: (1) To evaluate biochemical parameters in the blood after oral administration of *n-*hexane extract and 3′-demethoxy-6-O-demethylisoguaiacin (D) and norisoguaiacin (N) orally (2) to determine the stress biomarkers such as TBARS and glutathione peroxidase GSH-Px activity, and (3) to evaluate anatomopathological changes in organs such as the brain, liver, kidney, and lung after oral administration of *n-*hexane extract of *Ac* and D and N.

## Results

### Analysis of biochemical parameters

The effect of extract and lignans on biochemical parameters is depicted in Table 1. In the low-dose administered group of *n-*hexane extract of *A. cina*, the biochemical parameters as ALB, TP, GLO, Ca, BUN, AMY, ALT, TBIL, ALP, and CRE studied were found to be within the normal biological limits. The lignans at all four dose levels did not affect ALB, TP, GLO, Ca, BUN, P, AMY, ALT, TBIL, and CRE. Hepatic indicator profiles such as ALT and ALP were not significantly higher under the influence of doses. The kidney function parameters, like BUN and CRE levels, were average compared to those of the control group. The GLU levels were significantly affected with *n-*hexane extract, and with the lignans, the levels were significantly higher (p < 0.05). The *n-*hexane extract affected the phosphorus level at one point of the reference value (p < 0.05). The CHOL levels in animals treated with extract were affected with levels very high (p < 0.05). The CK levels were higher than the reference values, but in control groups, they were higher (p < 0.05).

**Table 1:** Biochemical parameters of rats treated orally after 14 days with *n*-hexane extract and lignans from *Artemisia cina.*

Treatments	Means ± standard deviation															
	ALB	TP	GLO	A/G	Ca	GLU	BUN	P1	AMY	CHOL	ALT	TBIL	ALP	CRE	BUNCRE	CK
*n-*Hexane extract of *Artemisia cina* (mg/mL)																
4	39.9^a^ ± 1.49	65.00^a^ ± 4.86	27.0^a^ ± 2.77	1.64^abc^ ± 0.42	2.79^ab^ ± 0.35	12.21^a^ ± 2.94	7.89^b^ ± 1.31	4.38^a^ ± 0.40	765.20^a^ ± 45.07	3.98^a^ ± 0.68	43.60^bca^ ± 8.29	3.7^a^ ± 0.53	363.20^ab^ ± 43.10	43.2^a^ ± 12.84	37.25^a^ ± 10.72	1299.7^a^ ± 607.87
40	38.7^a^ ± 2.07	52.5^bc^ ± 5.15	13.7^c^ ± 3.46	2.94^a^ ± 0.56	2.64^ab^ ± 0.17	11.7^ab^ ± 2.92	6.69^bcd^ ± 1.30	4.23^a^ ± 0.81	561.6^ab^ ± 90.24	3.68^ab^ ± 0.81	61.00^a^ ± 21.69	3.2^a^ ± 0.99	438.50^a^ ± 61.92	42.0^a^ ± 10.52	33.50^a^ ± 9.95	1374.0^a^ ± 885.30
240	37.32^a^ ± 2.40	57.96^ab^ ± 3.17	22.43^a^ ± 3.92	1.68^abc^ ± 0.43	2.80^a^ ± 0.23	9.96^abc^ ± 0.43	7.01^bc^ ± 0.76	3.77^ab^ ± 0.28	756.00^a^ ± 59.49	2.92^bc^ ± 0.24	53.00^abc^ ± 5.10	4.1^a^ ± 0.76	421.40^a^ ± 67.98	41.67^a^ ± 4.51	37.50^a^ ± 9.18	911.40^a^ ± 791.80
1600	36.82^a^ ± 3.15	58.18^ab^ ± 6.54	21.36^ab^ ± 3.60	1.74^abc^ ± 0.15	2.82^a^ ± 0.22	10.57^ab^ ± 1.27	7.15^bc^ ± 0.86	3.41^abc^ ± 0.27	418.00^b^ ± 45.65	2.96 ± 0.47	56.25^ab^ ± 8.18	3.7^a^ ± 1.07	351.25^abc^ ± 57.93	40.00^a^ ± 9.83	35.00^a^ ± 11.92	764.50^a^ ± 287.85
3′-Demethoxy-6-O-demethylisoguaiacin (1) and norisoguaiacin (2) (mg/mL)																
2	26.28^ab^ ± 15.32	53.83^bc^ ± 4.60	23.16^a^ ± 2.06	1.38^bc^ ± 0.39	2.21^a^ ± 0.18	6.53^c^ ± 1.19	11.25^a^ ± 0.83	2.67^c^ ± 0.58	406.75^b^ ± 53.35	2.53 ± 0.27	22.80^d^ ± 1.30	4.0^a^ ± 0.60	153.25^d^ ± 36.85	59.5^a^ ± 18.36	44.25^a^ ± 5.80	654.75^a^ ± 114.85
20	15.50^b^ ± 16.15	38.90^d^ ± 7.47	21.40^ab^ ± 5.16	1.32^c^ ± 1.21	2.20^b^ ± 0.41	6.41^c^ ± 2.05	5.99^bcd^ ± 0.86	2.52^c^ ± 0.54	370.25^b^ ± 40.20	2.13 ± 0.15	38.50^cd^ ± 4.65	2.65^a^ ± 0.74	308.00^abc^ ± 48.83	44.75^a^ ± 13.12	25.50^a^ ± 8.58	732.75^a^ ± 158.66
120	33.28^a^ ± 1.28	46.68^cd^ ± 1.70	13.40^c^ ± 1.27	2.50^abc^ ± 0.24	2.56^ab^ ± 0.14	7.76^bc^ ± 0.78	5.17^cd^ ± 0.55	2.79^bc^ ± 0.19	424.80^b^ ± 42.43	2.56 ± 0.13	39.40^bcd^ ± 3.51	3.31^a^ ± 0.46	381.75^ab^ ± 51.23	67.00^a^ ± 17.15	21.00^a^ ± 7.75	517.80^a^ ± 92.18
800	35.00^a^ ± 3.00	48.34^bcd^ ± 4.03	13.34 ± 1.23	2.62^abc^ ± 0.13	2.76^ab^ ± 0.13	10.63^ab^ ± 1.26	4.77^d^ ± 0.93	3.01^bc^ ± 0.29	438.40^b^ ± 66.25	2.42 ± 0.15	42.80^abc^ ± 4.60	2.69^a^ ± 0.29	346.00^abc^ ± 50.38	50.80^a^ ± 23.52	22.00^a^ ± 7.44	637.75^a^ ± 134.76
1600	36.23^a^ ± 1.59	49.80^bc^ ± 1.94	13.58^c^ ± 0.64	2.24^abc^ ± 0.98	2.50^ab^ ± 0.39	10.99^ab^ ± 1.24	5.63^cd^ ± 0.77	2.92^bc^ ± 0.51	382.00^b^ ± 36.03	2.39 ± 0.21	46.80^abc^ ± 8.61	3.41^a^ ± 1.05	253.60^bcd^ ± 30.66	66.50^a^ ± 21.25	22.75^a^ ± 9.54	591.20^a^ ± 113.04
Testigo	37.42^a^ ± 1.11	51.10^bc^ ± 1.77	13.90^c^ ± 0.59	2.76^ab^ ± 0.09	2.71^ab^ ± 0.12	11.17^ab^ ± 1.03	5.27^cd^ ± 0.52	2.95^bc^ ± 0.24	435.80^b^ ± 55.02	2.39 ± 0.15	44.80^abc^ ± 3.56	2.95^a^ ± 0.96	315.00^abc^ ± 53.35	57.00^a^ ± 11.43	22.75^a^ ± 4.79	536.00^a^ ± 66.67
K30 (1600 mg/mL)	31.08^ab^ ± 11.58	46.54^cd^ ± 7.48	15.46^bc^ ± 4.25	2.24^abc^ ± 0.97	2.49^ab^ ± 0.38	9.86^abc^ ± 2.73	5.59^cd^ ± 0.85	2.92^bc^ ± 0.51	360.60^b^ ± 57.12	2.39 ± 0.21	46.80^abc^ ± 8.61	3.41^a^ ± 1.05	203.30^cd^ ± 116.34	58.60^a^ ± 25.51	29.20^a^ ± 16.61	5810.20^a^ ± 127.21
Reference values	30–48	53–82	10–50		1.8–3.55	2.8–7.9	1.8–10.3	1.06–3.55	200–2500	0.5–2.4	20–61	0–12	20–61	18–71		48–520

Means within the same column with different literals statistically differ, p < 0.5.

### Anatomopathological changes of Wistar rats treated with *n-*hexane extract and lignans of *Artemisia cina*

The anatomopathological changes of Wistar rats treated with *n-*hexane extract and lignans of *Artemisia cina* are described in Table 2. Figures that describe the changes are in Figs. 1 to 25.

**Table 2:** Anatomopathological changes of liver, brain, lung, and kidney of Wistar rats treated with *n-*hexane extract and lignans of *Artemisia cina.*

Treatment	Brain	Liver	Lung	Kidney
*n-*hexane extract of *A. cina* 4 mg/Kg	No apparent pathological changes (NAPC)	Mild to moderate lymphocytic cholangitis Bile duct hyperplasia Moderate (Fig. 1)	Moderate interstitial pneumonia (Fig. 2)	Mild tubulonephrosis
*n-*hexane extract of *A. cina* 40 mg/Kg	Mild diffuse to multifocal neuronal degeneration Moderate gliosis. (Fig. 3)	Mild lymphocytic cholangitis Diffuse albuminous degeneration Moderate (Fig. 4)	Moderate to severe interstitial pneumonia (Fig. 5)	Mild tubulonephrosis (Fig. 6)
*n-*hexane extract of *A. cina* 240 mg/Kg	Moderate multifocal neuropil degeneration Moderate peripheral chromatolysis Severe multifocal neuronal degeneration in a rat (R3). Moderate gliosis Neuronophagy and satellitosis (Fig. 7)	Mild lymphocytic cholangitis Moderate to severe multifocal to diffuse albuminous degeneration Mild to moderate bile duct hyperplasia (Fig. 8)	Moderate to severe interstitial pneumonia (Fig. 9)	Moderate tubulonephrosis (Fig. 10)
*n-*hexane extract of *A. cina* 1600 mg/Kg	Moderate to severe multifocal neuronal degeneration. Severe diffuse neuropil degeneration Moderate to severe chromatolysis Moderate to severe gliosis Neuronophagy and satellitosis (Fig. 11)	Mild lymphocytic cholangitis Moderate to severe multifocal to diffuse albuminous degeneration Mild to moderate bile duct hyperplasia (Fig. 12)	Mild interstitial pneumonia. (Fig. 13)	Moderate tubulonephrosis (Fig. 14)
Lignans 2 mg/Kg	Degeneration of the neuropil Peripheral chromatolysis Neuronophagia and satellitosis Mild multifocal (Fig. 15)	Mild occasional multifocal lymphocytic cholangitis with eosinophils (R1) Moderate albuminous degeneration Mild bile duct hyperplasia	Severe interstitial pneumonia	Moderate diffuse tubulonephrosis (Fig. 16)
Lignans 20 mg/Kg	neuronal degeneration Mild to moderate diffuse neuropil degeneration Mild focal gliosis (R3) Neuronophagia and satellitosis (Fig. 17)	Mild multifocal lymphocytic cholangitis (R2) Moderate diffuse albuminous degeneration Bile duct hyperplasia Moderate (Fig. 18)	Severe interstitial pneumonia (Fig. 19)	Mild tubulonephrosis (Fig. 20)
Lignans 120 mg/Kg	Neuronal degeneration Mild to moderate multifocal Moderate multifocal to diffuse neuropil degeneration. Moderate peripheral chromatolysis Neuronophagy and satellitosis (Fig. 21)	Focal lymphocytic cholangitis a Mild multifocal Severe diffuse albuminous degeneration Moderate bile duct hyperplasia	Mild interstitial pneumonia	Mild tubulonephrosis (Fig. 22)
Lignans 800 mg/Kg	Mild multifocal neuronal degeneration Mild to moderate multifocal neuropil degeneration. Moderate focal gliosis, neuronophagy and satellitosis Severe peripheral chromatolysis (Fig. 22)	Mild multifocal lymphocytic cholangitis (R1) Moderate diffuse albuminous degeneration Mild to moderate bile duct hyperplasia (2 rats) (Fig. 23)	Moderate interstitial pneumonia	Mild to severe tubulonephrosis (Fig. 24)
Control	NAPC	Mild diffuse albuminous degeneration	Severe interstitial pneumonia	NAPC

**Figures 1 to 25:**
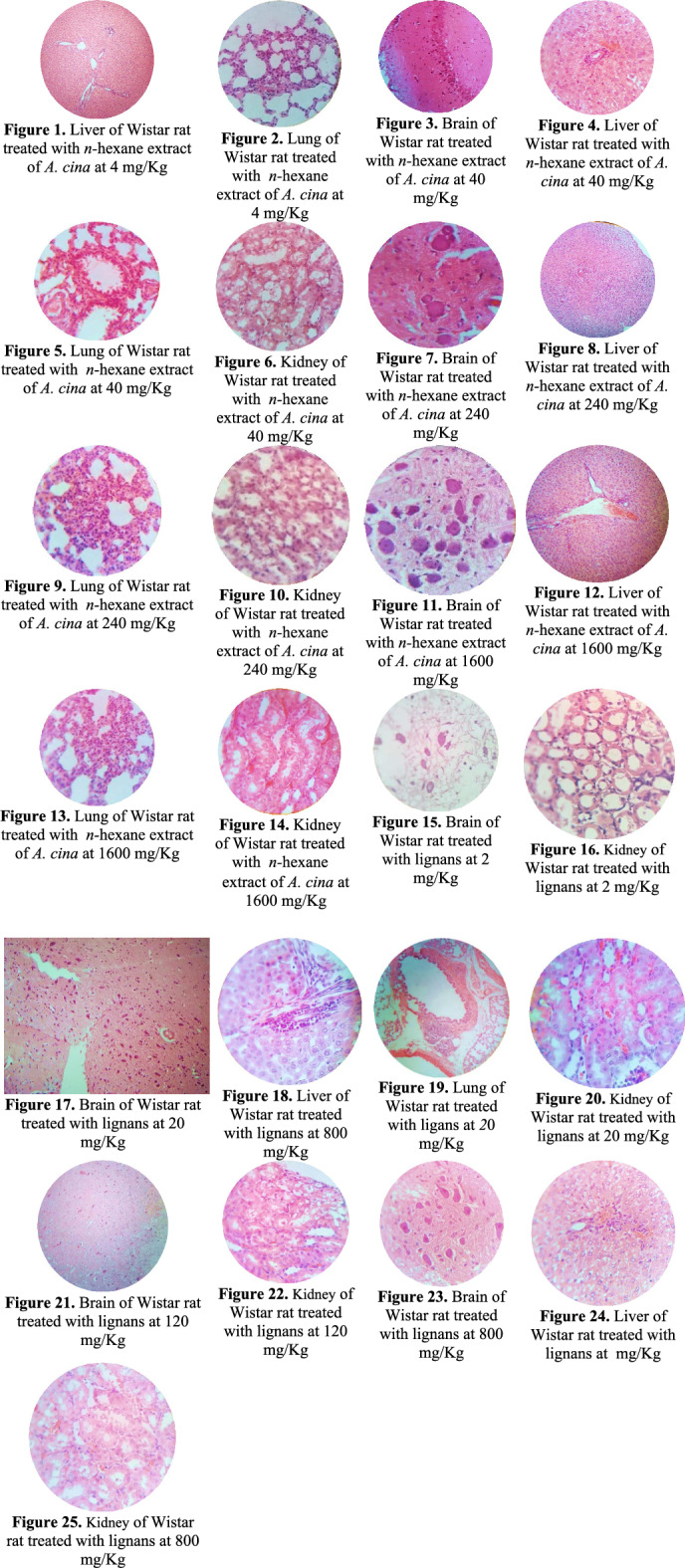
Image gallery for histopathological description of toxicological changes described in Table 2.

Table 2 describes the anatomopathological changes, and Figs. 1 to 25 contain the histopathological images. Fifteen samples from each organ corresponding to the five animals in each experimental group were analyzed and photographed using an optical microscope at 10X magnification.

For the histopathological interpretation, please review Table 2.

### Evaluation of oxidative stress biomarkers: TBARS and GSH-Px

**Figure 26:**
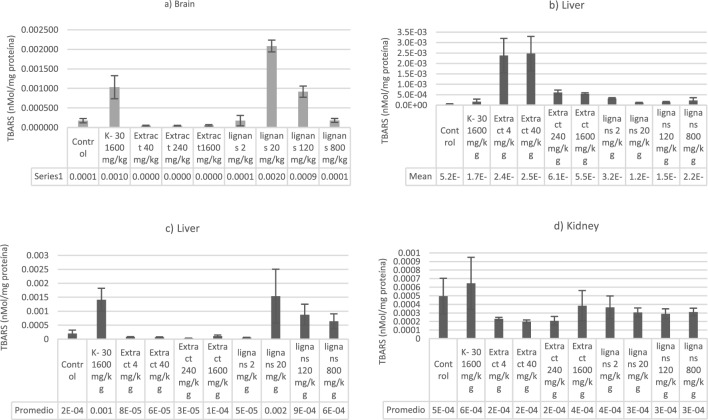
Quantification of thiobarbituric acid reactive substances (TBARS) values in (**a**) brain, (**b**) lungs, (**c**) liver, and (**d**) kidney of Wistar rats treated with *n-*hexane extract of *Artemisia cina* and 3′-demethoxy-6-O-demethylisoguaiacin and norisoguaiacin (lignans) orally treated.

**Figure 27:**
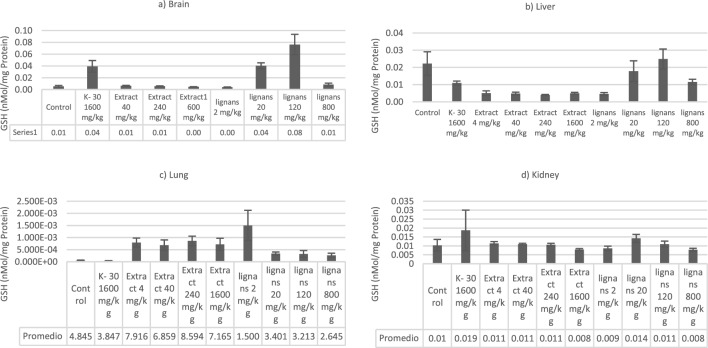
Quantification of glutathione (GSH-Px) values in (**a**) brain, (**b**) lungs, (**c**) liver and (**d**) kidney of Wistar rats treated with *n-*hexane extract of *Artemisia cina* and 3′-Demethoxy-6-O-demethylisoguaiacin and norisoguaiacin (lignans) orally treated.

In the post-weaning phase, animals receiving n-hexane extract and lignans, after 14 days, had a reduction in TBARS levels in some organs compared with control animals (Fig. 26). In general, the activity of antioxidant biomarkers like GSH of extract and lignans-fed rats increased (p < 0.05) compared with the control (Fig. 27). It is important to note that the enzyme activities did not all change concomitantly, nor did we observe effects in all experiments. We observed increased GSH values in the brain group with 20 and 120 mg/Kg lignans. The comportment was equal in the TBARS brain group (p < 0.05). In the liver groups, the GSH activity was increased while the doses were raised in the treatment with lignans. Moreover, the TBARS activity was higher in groups treated with 4 and 40 mg/Kg of n-hexane extract (p < 0.05). GSH activity in the lung demonstrated similar activity between extract treatments; however, lignan-treated rats demonstrated reduced activity (p < 0.05). GSH and TBARS in the kidneys of the rats treated with lignans and the extract did not show changes concerning the control groups.

## Discussion

In the biochemical parameters, cholesterol comes mainly from hepatocytes, intestinal mucosa, gonads, and adrenal glands; its increase may indicate a possible liver disease due to cholestasis or the onset of nephrotic syndrome, findings compatible with those found in histopathology. Alkaline phosphatase is present in the liver, bones, kidneys, and small intestine; the increases may be due to increased enzymes related to cholestatic liver diseases^19^. Phosphorus elevations may be due to renal failure or lysis by cell damage, which releases intracellular phosphorus^20^.

On the other hand, in the *n-*hexane extract at 1600 mg/kg, an elevation of cholesterol and alkaline phosphatase was found in the same way. According to the findings, the treatments with hexane extract of *A. cina*, the lowest and highest concentration, respectively, were where indications of kidney damage and liver damage mainly due to cholestasis are suggested^21^.

Kumar^22^, mentioned a clinical case where he reported cholestatic hepatitis with marked cholestasis and mild portal and lobular inflammation through a biopsy, referring that the patient ingested an herbal supplement containing artemisinin. Artemisinin and its derivatives are widely used as a herbal supplement to treat malaria; reports of severe liver injury secondary to antimalarial drug combinations containing artemisinin and its derivatives are rare. Osonuga et al.^23^ used rats to orally administer artemether (a methylated derivative of artemisinin), in which a significant elevation of ALT and AST (aspartate aminotransferase) was found.

In most treatments, an elevation of CK and Glucose, respectively, was found to be able to find said alterations due to the stress caused by the sacrifice method and obtaining blood and tissue samples, thus causing a break in the muscle fibers^24^. CK, or creatine kinase, is a cytoplasmic enzyme in cardiac and skeletal muscle cells^25^. Its elevation is due to multiple muscle trauma, persistent muscle trauma, ischemia, or inflammation^25^.

In general, the histopathological analysis reveals that groups 3, 4, and 8 had the greatest severity of lesions, especially in the brain, liver, and kidney.

In the brain, the microscopic findings were more evident and severe in groups 3 and 4, which coincides with high doses of the *n-*hexane extract of *A. cina;* neurotoxicity has been reported in plants of the genus Artemisia, associated with the compound artemisinin, among other terpenes^26^. Likewise, the lignans groups, especially those of group 8 (800 mg/kg), presented degeneration of the neuropil and peripheral chromatolysis. The lignans showed signs of toxicity (peripheral chromatolysis) from the 2 mg/kg dose, in contrast to *the n-hexane extract* observed from the 240 mg/kg dose.

In the liver, moderate multifocal hydropic degeneration was revealed, moderate multifocal lymphocytic and eosinophilic periportal hepatitis, and bile duct hyperplasia. Cases of cholestatic hepatitis have been reported, presumably associated with treatments based on Artemisinin^22^ and hepatotoxicity associated with terpenes such as sesquiterpenes^27^. Another compound associated with liver damage is camphor, an essential oil reported as causing hepatocellular damage, hepatomegaly, granulomas, and eosinophilic infiltrate^27^. The presence of eosinophilic inflammatory infiltrate can be generated in response to the toxic effect where the recruitment of eosinophils is triggered, which, through the segregation of IL-4, promotes the proliferation of hepatocytes^28^.

Although bile duct hyperplasia is frequently associated with poisoning, for example, with alkaloids, the literature reports that rodents can show “typical” bile duct hyperplasia related to old animals, in which the hyperplasia is in the triad porta, unlike intoxicated animals, which present "atypical" ductal hyperplasia, that is, the hyperplasia is multifocal^29^. In the present study, both forms of hyperplasia were found, mainly "typical”. This finding is taken into account since the hyperplasia of typical bile ducts is more associated with old animals, which does not agree with the age of the animals in the study; in addition, in cases of intoxication, “typical” bile duct hyperplasia can be found, together with the presence of cholangitis, it leads to the suspicion that there is a relationship between the treatments and bile duct hyperplasia. Said hyperplasia occurs when there is cholestatic hepatitis; another triggering factor is nodular regeneration from the liver as a consequence of damage^30^. Additionally, in group 3, one liver presented severe diffuse albuminous degeneration.

Two livers with mild to moderate albuminous degeneration were observed in the polyvinylpyrrolidone and control groups; however, K-30 has not been associated with hepatotoxicity. The control group showed mild diffuse albuminous degeneration, an unspecific praise.

Therefore, these findings were only found in some rats (2 in each group), without any other apparent lesion, which could be an incidental finding. Kidney tubulonephrosis was observed in all groups, with different degrees of severity, being more severe in the *n-hexane* extract and lignan groups compared to the control groups. The kidney is the primary organ that excretes many xenobiotics, representing a significant concentration of phytochemicals. It is an organ susceptible to hypoxia, so in the case of control groups, mild tubulonephrosis may result from management during sampling. Likewise, it is known that tannins can cause tissue destruction in the kidneys characterized by degeneration and acute tubular necrosis, reported in cattle^31^.

In the lung, all the groups presented different degrees of severity of interstitial pneumonia; however, when found with the same lesion pattern in control groups, this lesion is expected to be independent of experimental treatment; the inflammatory infiltrate was mainly composed of lymphocytes, and macrophages, occasionally eosinophils, neutrophils, and Mott cells were observed. The inflammatory responses can be associated with immune-mediated processes or hypersensitivity^32^. Also, the presence of interstitial pneumonia can be associated with viral infections, or it is known that rodents are susceptible to respiratory diseases often triggered by environmental factors such as sawdust, temperature changes, etc.^33^. Some xenobiotics administered by the nasal way cause interstitial pneumonia; in this case, the hyperplastic Club cells are occasionally presented. The present study observed them in some groups, including the control group. Club cells have multiple functions; among them, they contribute to the production of pulmonary surfactant, their high metabolic capacity contributes to detoxification processes, and they respond to certain stimuli by multiplying and differentiating from other cell types (hyperplasia). In some cases, the presence of bacillary bacteria was evidenced, although these were scarce so that they may be associated with sample management. The finding does not corroborate the treatment the animals were subjected to as the direct cause of pneumonia^34^.

According to the literature review on toxicity associated with plants of the genus *Artemisia*, terpenes are the compounds most associated with toxicity (embryotoxic, neurotoxic, hepatotoxic, allergenic, and genotoxic) among other plants^35,36^.

Various mechanisms combat the damage to the body caused by free radicals; among these mechanisms is GSH, which includes enzymes related to metabolism and helps maintain homeostasis^37^. GSH is constantly changing since it is found in the main organs responsible for homeostasis, such as the liver, kidneys, muscles, heart, and intestines. These changes in GSH concentration are widely used to measure oxidative stress^37^.

The graphs show that kidney GSH is homogeneous in most dosages, with polyvinylpyrrolidone at 1600 mg/kg and cinaguiacin at 20 mg/kg being the highest.

Aziz et al.^38^ evaluated the behavior of antioxidant defenses in chronic renal failure. They reported decreasing levels of GSH as the disease progressed, thus concluding that it was a response to increased oxidative stress.

The data obtained by an organ in the GSH tests can be graphically observed in the braincase as an increase in cinaguiacin at 20 and 120 mg/kg and k30 at 1600 mg/kg.

Consales et al.^39^ mentioned that GSH-Px synthesis in the brain is the same as in other tissues. However, this molecule is found uniformly throughout the brain, and the presence of precursor enzymes for its synthesis is reported, mainly in glial cells and neurons. When homeostasis is lost, the precursors for GSH-Px synthesis are obtained from the plasma; they cross the blood–brain barrier through specific transporters^39^. In the present study, the GSH was elevated; moreover, the TBARS were dismissed to explain that the extract and the lignans induce the synthesis of GSH as a possible protection agent for lipo-peroxidation.

The transport of GSH, as such, through specific transporters between astrocytes and neurons is a poorly studied process and requires further investigation^40^. It has been found that there is a close relationship between oxidative stress and neurodegenerative processes. Therefore, GSH-Px is crucial as it represents a line of defense against oxidative stress, which different enzymes use to improve or delay the damage caused by oxidative stress^40^. With the GSH elevations in the graphs and the quote mentioned above, we can suggest cinaguaiacine at 20 and 120 mg/kg and polyvinylpyrrolidone at 1600 mg/kg as neuroprotective.

### Glutathione peroxidase (GSH-Px)

Is a molecule that participates in cellular homeostasis, maintaining the defense against oxidative damage^41^. Oxidative stress is caused by increased reactive oxygen species (ROS), which are essential in cardiovascular diseases such as hypertension, cardiac hypertrophy, heart failure, and ischemia–reperfusion^41^.

In the liver, GSH increased in the control group, cinaguacin at 20 and 120 mg/kg. On the other hand, in TBARS, there was an increase in the hexane extract at 4 and 40 mg/kg. The liver is a vital organ involved in the synthesis and degradation of GSH; it has the highest concentrations of GSH because hepatic synthesis depends on the speed of GSH export to plasma, bile, and mitochondria through different systems^42^. Thus, a high concentration of GSH is related to the liver's primary function, detoxification^42^.

GSH synthesis occurs in the cytosol of cells from precursor amino acids and the action of different enzymes^43^. Different factors are involved in stimulating the synthesis of GSH; for example, insulin. In diabetic patients, a decrease in this hormone produces a reduction in erythrocyte GSH, resulting in oxidative stress in cells^43^. Another condition is the rapid growth of hepatocytes due to partial hepatectomy or after acute liver injury^43^.

In the case of the liver in TBARS, the elevations in the graph are very noticeable in the case of *n-*hexane extract at 4 and 40 mg/kg concerning the other groups. Similarly, in the case of the lung, the predominant elevations are polyvinylpyrrolidone at 1600 mg/kg and cinaguiacin at 20 mg/kg. Panche et al. state that flavonoids can prevent injuries caused by free radicals; these are oxidized by free radicals, which makes the radicals more stable and less reactive. In other words, the flavonoids will stabilize the reactive oxygen species^44^.

## Conclusion

*N-*hexane extract and *D* and *N* of *A. cina* did not have damage at therapeutic doses. *D*, *N*, and *n*-hexane extract of *A. cina* do not cause histopathological damage at pharmaceutical doses. However, the brain, kidney, and liver are related to biochemical parameters at higher doses. However, compounds are proposed as antioxidant agents. The dose for anthelmintic activity may be considered secure for oral administration in rats.

## Materials and methods

### Plant material

The *Artemisia cina* plant is for commercial use and was purchased in a state of prefloration from authorized laboratories (Hunab® laboratories, México). Q.F.B David Solórzano Moreno carried out quality control. Hunab® laboratories have a greenhouse and controlled growth conditions for *A. cina*.

A complete specimen was left in the herbarium of the Faculty of Higher Studies Cuautitlán (UNAM, México) for identification and registration under the supervision of Dr. Alejandro Torres-Montúfar. The voucher number was 11967. The identification and purchase voucher plant are in the supplementary material. *A. cina* is a typical specimen, then it is not part of the IUCN Policy Statement on Research Involving Species at Risk of Extinction.

### Elaboration of *n*-hexane extract of *Artemisia cina*

The leaves and stems of dry *A. cina* O. Berg ex Poljakov (Asteraceae) were macerated with *n-*hexane for 24 h. The plant was grown at 80% humidity, 24 °C temperature, and pH 6.3 soil. Chromatographic techniques separated the dry extract, and the lignans 3′-demethoxy-6-O-demethylisoguaiacin and norisoguaiacin were identified and isolated. The structure of molecules was confirmed by nuclear magnetic resonance^14^.

### Pharmaceutical dispersion of *A. cina*

The *n*-hexane extract and the lignans were lyophilized, and the dry dust was mixed with polyvinylpyrrolidone at a 1:1 proportion for pharmaceutical dispersion^45^.

### Wistar rats

Fifty male and female weaned Wistar rats were randomized into ten experimental groups. Rats were seven weeks old and had water and food ad libitum. The rats were treated under an experimental protocol established in the CICUAE (internal committee for the care and use of experimental animals). Also, all methods were performed according to the relevant guidelines and regulations by ARRIVE guidelines (Plos Bio 8(6), e1000412,2010). The n*-*hexane extract and lignans were administered orally to rats, and the behavioral changes and acute toxic signals were monitored. After fifteen days post-treatment, rats were euthanized following the Official Instructions for Animal Care (NOM-051-ZOO-1995, NOM-033-Z00-1995 y NOM-062-ZOO-1999, www.senasica.gob.mx). The euthanization method was the carbon dioxide chamber established by the official Mexican standard (NOM-051-ZOO-1995, NOM-033-Z00-1995, and NOM-062-ZOO-1999, www.senasica.gob.mx). Blood samples were taken by intracardiac punction and deposited in lithium heparin tubes. The Brain, lungs, kidneys, and liver were removed, and two samples were taken for histopathological studies and oxidative stress biomarkers in buffered formalin at 10% and lysis solution, respectively.

### Experimental design

The treatment was administrated orally following the 420 guide from OECD (Guideline for testing of chemicals) for acute oral toxicity evaluation^46^. Fifty rats at seven weeks old were randomly distributed in ten experimental groups. The following groups were administered oral *n-*hexane extract of *A. cina*: Group 1: 4 mg/Kg; Group 2: 40 mg/Kg; Group 3: 240 mg/Kg; Group 4: 1600 mg/Kg. The following groups were administered Lignans (cinaguaiacin) orally: group 5: 2 mg/Kg; group 6: 20 mg/Kg; group 7: 120 mg/Kg; group 8: 800 mg/Kg. Groups 9 and 10 were controlled with distilled water and polyvinylpyrrolidone at 1600 mg/Kg. Rats were observed every four hours after treatment for 14 days. The behavioral changes were documented, and the mortality percentages were made.

### Analysis of biochemical parameters

After 14 days of treatment, the rats were euthanized, and the intracardiac punction took the blood. The samples were deposited in lithium heparin tubes, and the analysis was made immediately. Albumin (ALB), total proteins (TP), globulins (GLO), total bilirubin (TB), alanine aminotransferase (ALT), alkaline phosphatase (ALP), creatine kinase (CK), amylase (AMY), triglycerides (TG), cholesterol (CHOL), glucose (GLU), creatinine (CRE), ureic nitrogen (BUN), calcium (Ca) and phosphorus (P) were measured with blood chemistry analyzer (Celercare V5 – MNCHIP, KABLA®)^47^.

### Evaluation of anatomopathological changes

The liver, brain, lungs, and kidneys of all animals were dehydrated with increasing alcohol content (incubation in 70, 80, 90, and 96% twice and in 100% alcohol three times for 30 min each one, submitted to clearing in xylol (2 incubations of 2 h each), then they were embedded in histological blocks of paraffin (2 incubations of 2 h each in an oven at 60 °C). The blocks were refrigerated and sectioned at 5.0 μm with a Scientific Instruments microtome, model 820 rotary. The sections were stained with hematoxylin and eosin and analyzed under an optical microscope.

### Evaluation of oxidative stress biomarkers: TBARS and GSH-Px

100 mg of liver, brain, lungs, and kidney tissue were weighed and mixed with lysis buffer. The samples were centrifuged at 13,000×*g* for 15 min at 4 °C. The supernatant was used for GSH-Px and TBARS tests.

### Tissue quantification GSH-Px

The quantification was made as reported by^48^ when the reaction of GSH/GSSG in the sample with acid 5,5′-dithiol-bis(2-nitrobenzoic) (DTNB) produced GS-TNB and TNB (acid 5-tio-2-nitrobenzoic) with a yellow color that spectrophotometric methods can measure at 400 to 430 nm. 200 μL of sample supernatant was added to 10 μL of acid sulfosalicylic at 5% (SSA). The samples were incubated for 15 min in freezing and centrifugated at 13,000 rpm for 15 min at 4 °C. The supernatant was recovered and added to the 800 μL reaction solution (S-Rx) of GSH. The reaction was incubated at 37 °C for 30 min. The reading was done at 405 nm with a spectrophotometer. A standard graph was made with known GSH concentrations.

### TBARS quantification

250 μL of supernatant was added to 250 μL of perchloric acid, and the samples were frozen for 15 min and then centrifuged for 13,000×*g* for 15 min. The supernatant was recovered, and 500 μL of Tiobarbituric acid was added at 0.67%. The mixed let was incubated at 90 °C for 60 min. Finally, the reading was done by spectrophotometer at 532 nm. A standard graph was done with malon-aldehyde known concentrations^49^.

### Statistical analysis

Data were analyzed by ANOVA using a completely randomized design with general linear proceed (PROC GLM) of SAS statistical packed (version 9.0). p < 0.05.

### Ethical statement

Comité Interno para el Cuidado y Uso de los Animales en Experimentación (CICUAE-FESC) approved the experimental protocol for Wistar rats under protocol number C22_06 on November 29, 2022. Rats were euthanized following the Official Instructions for Animal Care (NOM-051-ZOO-1995, NOM-033-Z00-1995, and NOM-062-ZOO-1999, www.senasica.gob.mx). All methods are reported by ARRIVE guidelines (https://arriveguidelines.org).

### *Artemisia cina* plant statement

The study of *the A. cina plant was carried out* by relevant institutional, national, and international guidelines and legislation in Mexico. The plant is cultivated, and therefore, the collection permit does not apply. *A. cina* is a plant that is not and will not be under the risk category of the UICN or any other conservation management organization (CITES Ó NOM 059).

## Data Availability

The databases used and analyzed during the current study are available from the corresponding author upon reasonable request.
